# On the effectiveness of multi-feature evacuation systems: an agent-based exploratory simulation study

**DOI:** 10.7717/peerj-cs.531

**Published:** 2021-05-19

**Authors:** Kashif Zia, Umar Farooq, Muhammad Shafi, Alois Ferscha

**Affiliations:** 1 Faculty of Computing and Information Technology, Sohar University, Sohar, Oman; 2 Department of Computer Science, University of Science and Technology Bannu, Bannu, Khyber Pakhtunkhwa, Pakistan; 3 Institute of Pervasive Computing, Johannes Kepler University, Linz, Austria

**Keywords:** Crowd evacuation, Agent based modeling, Rationality vs. emotionalism, Social influence, NetLogo

## Abstract

Evacuation modeling and simulation are usually used to explore different possibilities for evacuation, however, it is a real challenge to integrate different categories of characteristics in unified modeling space. In this paper, we propose an agent-based model of an evacuating crowd so that a comparative analysis of a different sets of parameters categorized as individual, social and technological aspects, is made possible. In particular, we focus on the question of rationality vs. emotionalism of individuals in a localized social context. In addition to that, we propose and model the concept of extended social influence, thereby embedding technological influence within the social influence, and analyze its impact on the efficiency of evacuation. NetLogo is used for simulating different variations in environments, evacuation strategies, and agents demographics. Simulation results revealed that there is no substantial advantage of informational overload on people, as this might work only in those situations, where there are fewer chances of herding. In more serious situations, people should be left alone to decide. They, however, could be trained in drills, to avoid panicking in such situations and concentrate on making their decisions solely based on the dynamics of their surroundings. It was also learned that distant connectivity has no apparent advantage and can be ruled out while designing an evacuation strategy based on these recommendations.

## Introduction

Emergency evacuation is often associated with crowd panic, which sometimes turns into a catastrophe. Usually, it happens due to irrational behavior based on factors such as fear, pain, suffocation, or hassle of just a few emotionally intense individuals in the crowd. Disturbance initiated by a small group of individuals transforms into herding, which might cause injuries and deaths (Zia et al., 2016). It is often hard to find empirical evidence for such incidents in terms of factors resulting in them. An experimental examination is generally considered dangerous and unethical. Therefore, it is a normal practice to utilize simulation for modeling and investigation of such systems. Many modeling methodologies have been adopted for conducting evacuation studies in the literature (Pelechano & Malkawi, 2008).

One way of modeling such systems focuses on considering global flows (Cova & Johnson, 2003) while the other concentrates only on local interactions (Yuan & Tan, 2007). The majority of practitioners advocate the use of latter (**individual-based models**) over the former (**flow-based models**), as it is believed that *a global outcome is what emerges out of local interactions, which could be unexpected most of the time and must be dealt with accordingly*. However, the understanding of the emergence of global patterns in relation to rules of local decision-making of individuals poses more questions than answers. Furthermore, other factors related to population, environment, and information must also be considered due to their obvious relevance.

Ideally, an evacuation strategy should utilize all exits, in such as a way that the distribution of population across the exits is well balanced in terms of time, which makes it a perfect flow control application (Helbing, Farkas & Vicsek, 2000; Helbing, Johansson & Al-Abideen, 2007). Evacuation models have been proposed focusing only on globally scaled collectives (Von Schantz & Ehtamo, 2015) such as clogging (Seyfried et al., 2009), bottlenecks (Kretz, Grünebohm & Schreckenberg, 2006) and faster-is-slower (Garcimartín et al., 2014) effects. However, modeling of an evacuation situation solely based on physics has been greatly criticised (Reilly, Dillon & Guikema, 2018; Batty, 2001; Radianti et al., 2013). The reason, as stated above, is that individual and localized (social) decisions happening at a local scale manifest into global patterns (Kania & Kramer, 2013). Therefore, at an individual level, an agent must be considered as a social entity (Schadschneider et al., 2011), instead of a particle, having its own preferences, biases and emotions, and capability to adapt according to the changing dynamics of the environment. Therefore, more and more researchers have started using agent-based models for evacuation simulation  (Wagner & Agrawal, 2014; Almeida, Rosseti & Coelho, 2013).

Since people are the central entities in these situations, it is, therefore, important to represent them reasonably well. However, it is hard to get the evidence of human behavior, particularly, in life-critical situations like evacuation. Therefore, in most of the cases, the questions about the efficiency of egress from a troubled environment are answered using behavioral theories and patterns observed in other domains such as crowd mobility, navigation, and finding ways. In addition to that, the dimensions across which such an inquiry can be extended are numerous including physical modalities (such as smoke and visibility, building design, signage, and exits’ designs), crowd dynamics (such as crowd density, lanes and flows, and population diversity), information (such as vision and voice, ICT enabled infrastructure, and the availability of information through social network or local connectivity), individual decision-making (such as emotions, rationalism, and panic behavior), and collective effects (such as leader-following, herding effect, and peer effect). This huge set of aspects could be classified under *individual*, *social* and *informational* domains. Since, most research in evacuation simulation is done at a limited scope, a combinatorial model meaningfully combining these aspects is a challenge and focus of this paper.

Zia & Ferscha (2020) addressed this challenge by developing a common agent based model combining individual, social and technological aspects. The model was tested on a grid based hypothetical environment with an aim of getting an insight into the effectiveness of a multi-feature evacuation strategy. Their model, however, has an apparent inconsistency as it transmits the status information of exits to agents using spatial features of the environment instead of social spread. The model proposed in this work helps coping this issue by replacing spatial spread of information based on a mechanism of *pure* social spread, thus, exploiting a social network of people. The proposed model also explores the impact of long-distance relationships, thereby introducing a new set of exit strategies, tested in an environment of two exits and having different geometric variations (representing complexity).

The selection of modeling methods certainly depends on modeler choice and modeling requirements. Agent Based Modeling ABM, (Macal, 2016; Wilensky & Rand, 2015) is one of the most promising techniques for modeling and simulation, which is capable of handling all the multi-facet and multi-layer complexity of crowd panic and evacuation systems. Salient features of (ABM) such as autonomy, re-activity, pro-activity and social interaction make this method a natural choice for scenarios requiring autonomous and adaptive participating agents (Liu, Jiang & Shi, 2016) under the influence of individual, social and informational aspects.

With ABM, we started developing models of evacuation simulations based on purely physical acpects (Ferscha & Zia, 2009), which were, later on, extended to incorporate cognitive aspects of human thinking (Zia et al., 2011). Social aspects and neighborhood influence were, then, integrated into our models (Sharpanskykh & Zia, 2014). Furthermore, we developed decision-making models based on game-theoretic approach (Zia et al., 2016). In our most recent work, we investigated the generation and spread of information from exits  (Zia & Ferscha, 2020). This paper contributes by proposing a model extension that:

1. removes the inconsistencies of the model proposed in  Zia & Ferscha (2020), where the spatial nature of information spread is replaced by a pure social spread.
2. introduces a new behavior in agents’ decision-making repertoire, where the agents are more likely influenced by potentially long-distance ties, when their mobility is hindered.

The incorporation of a diversified but related set of features, in a combinatorial way, is itself an achievement, however, the novelty of this work comes from the inclusion of network structure, which enables us to exploit informational flow for the benefit of evacuees. In particular, it would explore the conditions which lead to an efficient evacuation with the help of social structure similar to real life. In other words, this work integrates a new model of social influence with other persisting aspects of interest. The proposed social model is anchored onto the small world phenomenon and the fact that novel information is expected to originate from long distant (or weak) ties, instead of, more frequently encountered strong ties. Moreover, Our work focuses on the question of rationality vs. emotionalism of individuals in a social context while focusing on modeling local interactions. This phenomenon is well researched in social, psychological, and economics domains but relatively less explored in the domain of modeling and simulating evacuation scenarios. This work helps fill this research gap.

The rest of the paper is organized as follows. ‘Background and Motivation’ presents the literature review and the contribution of this paper. ‘Models’ provides a detailed description of models including the underlying space and time model. It also presents the decision-making model, which considers individual, social and informational aspects, in detail. The simulation structure and flow using the Overview, Design concepts and Details (ODD) protocol is presented in ‘ODD Protocol’ while ‘Simulation and Results’ is devoted to simulation setup and results. A detailed discussion on the results is presented in ‘Discussion’ while ‘Conclusion’ concludes the paper.

## Background and Motivation

Modelling human behaviour and devising efficient evacuation schemes have been a focus of research, now, for quite some time. Various literature surveys have been carried out summarizing and critically analyzing crowd evacuation models from different perspectives  (He et al., 2013; Vermuyten et al., 2016; Bellomo et al., 2016; Shiwakoti, Shi & Ye, 2019; Koponen & Nousiainen, 2016; Zollman, 2012; Murase et al., 2014; Fu, Shi & Wang, 2015; Zheng & Cheng, 2011).

ABM has been used extensively in the literature to implement and simulate evacuation scenarios. Researchers have studied the evacuation scenario of crowds in buildings, cities, and classrooms. Some studies presented in  Koponen & Nousiainen (2016); Zollman (2012); Murase et al. (2014) have investigated evacuation schemes from the social network aspect while others have studied them using nature-inspired theories and algorithms (Fu, Shi & Wang, 2015; Zheng & Cheng, 2011). Researchers in  Wang et al. (2015); Tanimoto, Hagishima & Tanaka (2010); Lo et al. (2006); Altshuler et al. (2005); Wang et al. (2016) have described crowd behaviour and evacuation schemes in detail. Out of this plethora of efforts, we detail a few which are more relevant to our model.

Li et al. (2016) studied crowed behaviour in metro using ABM. They studied passengers behaviour in stairs, ticket gates as well as pathways. The goal was to find a safer and faster way to evacuate passengers from metro in case of an emergency. They simulated various evacuation scenarios against different parameters and found that it took an optimal time of three minutes and eight seconds to evacuate all passengers without any injury. However, this goal was achieved with an increased number of ticket gates and the width of the stairs. Game theory was utilized in developing the evacuation model, as passengers think before they move and distribute to different locations. Liu, Jiang & Shi (2016) carried out a detailed study on effectively planning evacuation routes in highly populated buildings such as movie theaters and schools/colleges. They simulated evacuation schemes with four different layouts of classrooms. Changes in the layout of a classroom had an impact on evacuation routes. The students were categorised into two groups, in which one group was provided evacuation instructions while the other was left without any evacuation instructions. The former group of students performed better during the evacuation process compared with the latter group, as they followed quicker and safer evacuation routes. It was also learnt that classrooms with two exits were better for evacuation in an emergency. The overall framework of our model is inspired by the concepts taken from these two papers.

Moreover, many research activities come under the domain of ABM such as structural dynamics, agents’ characteristics and decision-making mechanisms, social behavior, and informational flow based on social ties. However, all these efforts are isolated in nature with respect to different aspects of interest. The model, presented in this work, integrates these aspects, which is important to study such scenarios in detail. It is inspired by the work of  Wang et al. (2015) and its extension presented in our previous work (Zia et al., 2016; Zia & Ferscha, 2020). Part of our proposed research is also inspired by the work presented in  Koponen & Nousiainen (2016), which has demonstrated the impact of appreciation on social interaction and strengthening relationships.

The model presented in  Wang et al. (2015) differentiated and analyzed the emotional and rational behaviors of agents among a given population. It exploited the generally observed behavior during violent evacuation scenes, represented as emotional behavior that mimics others and the completely opposite rational behavior. The model, we proposed in  Zia et al. (2016), questioned Wang et al. (2015) notion of rational behavior and introduced the idea of panic with a hypothesis that a rational agent only, in panic, would do the opposite of mimicking others. It incorporated a formal spatial model with the homophily based social network to capture the panic situation. This model, however, considers only individual characteristics of the evacuees and, thus, the decision about a chosen exit is based on the dynamics of a limited neighborhood. In Zia & Ferscha (2020), we extended this model and incorporated environmental dynamics in it. This extended model realized environmental dynamics using the potential field concept, a collective of the mechanisms used to capture exit events, dealing with *content*, content *spread*, and its *influence* on agents.

The extended model, presented in  Zia & Ferscha (2020), actually, adopted the concept of guided assistance. In our earlier work, we used a device named, LifeBelt, to provide vibrotactile assistance to the users wearing it, for guiding them towards the safe exit(s) (Ferscha & Zia, 2009). This device is particularly helpful in environments with poor visibility due to blackout or smoke, a potential situation, which might occur during a natural hazard or a technological failure (Ferscha & Zia, 2010). The device was able to actuate appropriate vibra elements based on what was sensed about flow at different exits. It was, thus, producing the locomotive behaviour (short-range mobility) in response to combined effect of directional guidance and social pressure in the neighbourhood. Extensive experimental trials helped us develop models of mobility in the presence of social and cognitive load during evacuations in ambient assisted environments (Zia et al., 2011). The developed model has enough fertility and it was used for city-scale evacuations (Zia & Mitleton-Kelly, 2013). It was also experimented in an industrial setup (Murauer et al., 2019).

Reiterating again, in the model presented in  Zia & Ferscha (2020), the status information of exits is transmitted to the agents using spatial features of the environment, namely: the potential field. Though, a physical environment, in the current technological advanced era, is certainly capable of transmitting such information. However, this cannot be considered as the social spread of information. To cope with this inconsistency, the model proposed in this paper uses only social spread through people. Also, this paper introduces a new behavioral capability, which tries to capture the strength of long-distance, weak ties supported by the concept of a small-world network, hoping that new information would most probably originate from unfamiliar/unknown corridors.

## Models

This section describes various models and the decision-making strategies used in this work.

### Space and time model

This work uses a two-dimensional hypothetical square-shaped simulation world that comprises equal size cells. It is a 51 × 51 cells world, in a grid form, having a total of 2,601 cells, where each cell is represented by an xy-coordinate point. Mobile agents reside on top of cells and they are capable of using the information available at their underlying cells and that of neighboring cells and/or agents, in making their decisions. A neighboring agent can be an agent in the proximity as well as at a distance. Simulation operates in a discrete manner and every agent gets its turn at each timestamp. Agents use incremented state of the world and, thus, make their decisions sequentially. The built-in engine of NetLogo (Wilensky & Rand, 2015) (the software used for simulation in this work), however, provides a fair chance to the agents based on random sequencing at each timestamp.

**Figure 1:**
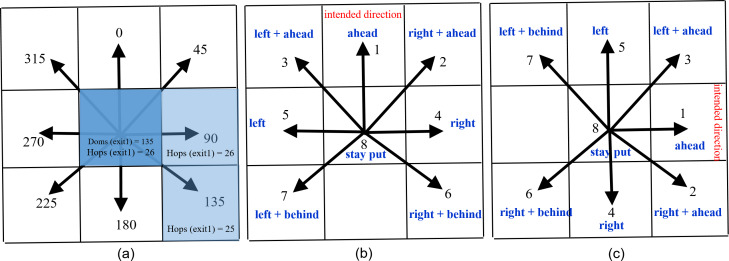
(A) Spread of floor field. (B), (C) Next cell selection mechanism. The textual descriptions of directions assume an agent at the central cell whose current heading (intended direction) is towards the cells at angle labelled as *ahead*. This diagram also shows Moore’s Neighborhood of size 1 of the cell at the center.

Each simulated space is initially setup with a floor field. As a result of the spread of the floor field, the state variables *Hops*—having the shortest distance (in terms of hop count) for each exit—and *Doms*—having the direction towards each of the exits corresponding to the shortest distance—of cells (Netlogo’s patches) are set. For a cell’s neighborhood, we have used Moore’s neighborhood model (Kretz & Schreckenberg, 2007), a cluster of nine neighboring cells around a cell. Figure 1A shows the neighborhood and directions for the cell in the center, exactly the same as used in NetLogo. The spread of floor field starts from exits and disperses outwards following a neighborhood based interaction. When a cell receives information from one of its neighbors, it checks its hop counter of the exit from where it originated. If the new value of hops is smaller than the current value held by the cell, the old value in Hops (for that exit) is replaced by the new value. The direction in Doms (for that exit) is also set to the direction towards the source cell. For example, in the situation shown in Fig. 1A, the information about exit1 to the cell in the center comes from cell at the right and behind and cell at the right. Since the hops to exit1 reported by the cell at the right and behind is less than what is reported by the cell at the right, direction to reach to exit1 is 135 (towards the source cell) and hop count is 1 more than the source cell.

**Figure 2:**
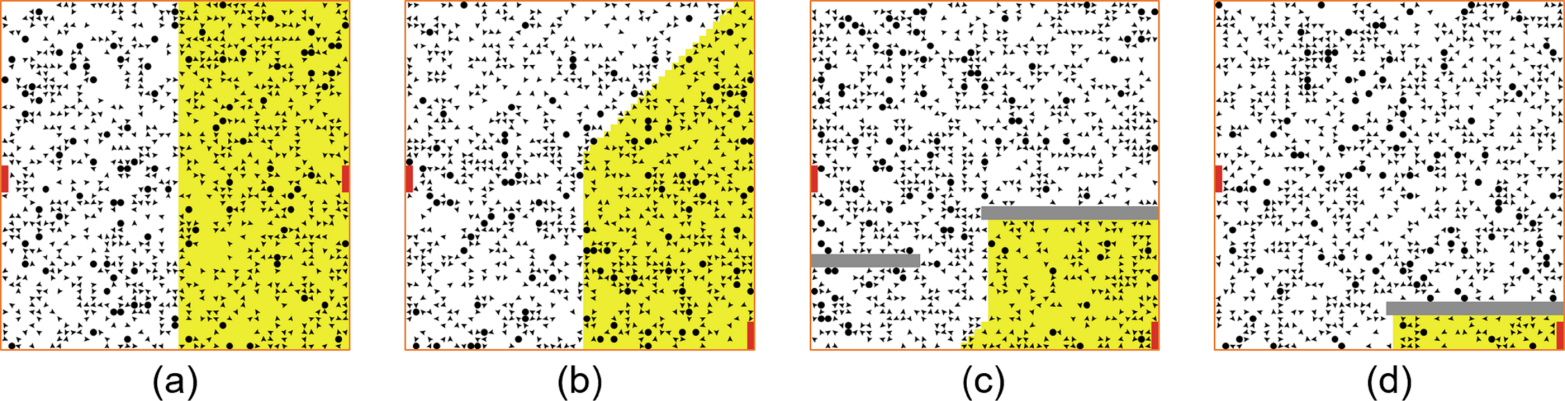
Four geometric variations of two exits shown in red with increasing unevenness between exits in terms of the nearness of patches to the exits from (A) to (B) to (C) and to (D) due to obstacles (in gray). The yellow patches are close to the exit on the left and the white patches are close to the exit on right.

The square-shaped simulation world (as shown in Fig. 2) has two exits. The figure also shows the geometric variations for the same two exits. These distributions show an increasing unevenness between exits in terms of the nearness of patches to the exits. Description of different types of patches is needed to completely understand the environmental structure. The patches could be:

1. Normal: A normal patch is walkable and its type is floor, as illustrated as a green patch in Fig. 2.
2. Obstacles: The patches that act as obstacles. The floor field spread does not pass through them and Doms and Hops of these patches, therefore, would have the default values (each −1). Obstacle patches are not walkable and they have type obstacle. Obstacles are shown as gray patches in Figs. 2C and 2D.
3. Exits: The patches that are used as exits. The floor field spread starts from these patches and, therefore, Doms and Hops of these patches have default values (each −1). Exit patches are walkable and they have type exit. These are shown red in Fig. 2.

This work uses the microscopic next cell selection strategy based on the concept of Moore’s neighborhood (Kretz & Schreckenberg, 2007). This strategy is about avoiding agents occupying the same cell. An agent chooses one of its eight neighbouring cells depending on the direction, in which it is moving. However, if the desired cell is already occupied, the agent adopts a strategy to choose an unoccupied cell. Figures 1B and 1C shows a dial towards the intended direction labeled with ‘1’. If the cell in this direction is occupied, the agent will choose the direction labeled with ‘2’, and so on. This strategy is based on empirical evidence from the work of Ferscha & Zia (2009).

### Decision making strategies

The decision-making process allows the agents to choose one of the two exits based on the following four strategies:

1. Strategy 1 (S1): which selects the nearest exit. It is based on **individual** aspect only.
2. Strategy 2 (S2): which selects the exit under the influence of local neighbors. It is based on localized **social** influence.
3. Strategy 3 (S3): which selects the exit under the influence of exit potential. It is based on **technological** influence.
4. Strategy 4 (S4): which selects an exit under the influence of both local and distant neighbors. It is based on small-world **social** influence.

These strategies are built upon the space and time model presented before and all of them except S1 support both emotional and rational types of agents. S1 acts as a base case, in which all agents proceed towards the nearest exit irrespective of type. In S2, only rational agents are influenced by a localized social circle. S3 uses exits’ potential along with the localized social circle. A comparison of the strategies S1, S2, and S3 is presented in Zia & Ferscha (2020). However, S3 described in Zia & Ferscha (2020) is updated in this work, and the updated version of this strategy transmits exits’ status information through agent-to-agent communication. The potential field of exits’ still remains a spatial feature, however, the spread in revised S3 is performed by agents instead of patches. S4 is an extended version of strategy S2. S4 has introduced a new behavioral capability, which attempts to capture the strength of long-distance weak ties supported by the concept of a small-world network. Thus, a distant agent, who is successfully moving would help a rational agent in panic. The following subsections describe these strategies in detail.

#### Strategy 1 (S1)

All agents in S1 proceed to the nearest exit, which is one of the two exits having a minimum value of the parameter *Hops*. This information is available at each patch, which is calculated using the floor field spread mechanism when the simulation setup is executed. This is a static strategy and used to compare it with the remaining strategies that allow dynamic changes between exits.


 
_______________________ 
 Algorithm 1: The Logic behind Strategy 2 (S2)                                ____ 
    Agent A is making a decision 
     Agent N is a random local agent 
     Ae ← current exit of A 
     Ap ← current panic index of A 
     Ne ← current exit of N 
     if A is emotional then 
         if Ae! = Ne then 
       if Ap > 0 then 
   Ae = Ne [with probability 0.1] 
   end 
   end 
    end 
    if A is rational then 
         if Ap <= 0 then 
       if Ae == Ne then 
   Ae = Aalternate [with probability 0.1] 
   else 
   Ae = Aalternate [with probability 0.9] 
   end 
   else 
       if Ae == Ne then 
   Ae = Aalternate 
   end 
   end 
    end    


#### Strategy 2 (S2)

The strategy S2 is based on game theory. Player 1 is the agent, who is making a decision while Player 2, a random neighbour from Moore’s neighbourhood, is the influencer. The logic behind S2 is implemented in Algorithm 1, where *A*_*e*_ represents the current exit of agent A, *N*_*e*_ represents the current exit of neighbor N, and *A*_*p*_ shows the panic index of agent A.

If the behaviour of an emotional agent is the same as the neighbouring agent, then, the payoff is maximum and, thus, the agent retains its behaviour and continues moving towards the current exit. The payoff remains maximum, even, when the behavior of random neighbor is the opposite of the agent behavior. This is the reason that these agents are “emotional”. However, there are 10% chances that an agent may change its behavior, if it is in panic, which ensures a random deviation. This means that the game is not really played by emotional but rational agents.

The strategy for a rational agent is dependent on whether it is in a state of panic or not. If an agent is not in a panic, it will be a source of strengthening its belief on current behavior, when the randomly selected neighbor has the same behavior. This case is represented as a maximum payoff. However, there are 10% chances that the agent may change its behavior, thus ensuring a random deviation. On the other hand, it will be a source of weakening an agent’s belief on the current behavior, when the randomly selected neighbor has the opposite behavior. This case is represented by a minimum payoff. However, there are 90% chances that the Agent may change its behavior leaving 10% chances for random deviation.

If the rational agent is in panic, we argue about entirely opposite behavior, in which the same behavior represents the minimum payoff. This directs the agent to change its behavior but strictly this time. Similarly, if the behavior of the randomly selected neighbor is different from the agent’s own behavior, the agent will strictly retain its recent behavior as no change of behavior is required in this case.

 
_________________________________________________________________________________________________ 
  Algorithm 2: The Logic behind Strategy 3 (S3)                                ____ 
    Agent A is making a decision 
     Agent N is a random local agent 
     Ae ← current exit of A 
     Ap ← current panic index of A 
     Ne ← current exit of N 
     PVL ← Potential of left exit at the current patch 
     PVR ← Potential of right exit at the current patch 
     Ep ← the exit with more potential among the above two 
     if A is rational and Ap <= 0 then 
         if Ae == Ne then 
       if Ae! = Ep then 
   Ae = Aalternate [with probability 0.25] 
   end 
   else 
       if Ae == Ep then 
   Ae = Aalternate [with probability 0.25] 
   else 
   Ae = Aalternate [with probability 0.50] 
   end 
   end 
    end 
    if A is rational and Ap > 0 then 
         if Ae == Ne then 
       if Ae == Ep then 
   Ae = Aalternate [with probability 0.50] 
   else 
   Ae = Aalternate [with probability 0.75] 
   end 
   else 
       if Ae! = Ep then 
   Ae = Aalternate [with probability 0.75] 
   else 
   Ae = Aalternate 
   end 
   end 
    end    

#### Strategy 3 (S3)

The decision taken by an agent using strategy S3 also depends on the agent being in panic or not. This model is only applicable to the rational agents, and the emotional agents will keep following the nearest exit. S3, however, is based on two additional conjunctures, regarding the spatial change, presented in Tan, Hu & Lin (2015). The spatial change could have a negative or positive effect. The effect is negative when detouring occurs due to the lack of knowledge. On the other hand, when exit utilization is balanced, the spatial change has a positive effect.

S3 (like S2) is based on systematic rational thinking, in which the factors of panic, social influence, and exits’ potential plays a major role in deciding an exit. Panic is given more weight (0.5) than the social influence and exits’ potential, which are assigned 0.25 weight each. Social influence and exits’ potential based on positive or negative role both decide these weights. For example, if an agent is not in panic (represented with a weight ’0′) but having a negative input of social influence (0.25) and having a negative input of exits’ potential (0.25), would result in a total weight of 0.50—showing the probability of switching of exit for the agent.

The logic behind S3 is given in Algorithm 2 and it is explained as follows. The current behavior of a rational agent A, in terms of left or right exit, is represented as *A*_*e*_. Agent A chooses a neighbor N, whose current behavior is represented as *N*_*e*_. The relative potential of the patch underneath the agent A is represented as *E*_*p*_. Since there are two exits, the value of *E*_*p*_ would be either left or right. The exit selection is based on the greater value among the two for left and right, which represent a more influential exit.

When A is not in panic, and *A*_*e*_ is equal to *N*_*e*_, then, the agent is considered quite satisfied. The agent has nothing to worry about when *A*_*e*_ also equals *E*_*p*_ and no change is needed in this case. However, if *A*_*e*_ is not the same as *E*_*p*_, then, the agent has something to worry about but two factors (no panic and neighbor’s influence) are still in its favor. Hence, it would only make a change in the exit plan with a 25% probability. When A is not in panic but *A*_*e*_ is not equal to *N*_*e*_, then, the agent A has something to worry about. The agent is satisfied, if *A*_*e*_ is the same as *E*_*p*_, as its own behavior and exits’ potential are both pointing towards the same exit. However, the negative neighbor’s influence would force the agent to switch to the other exit but only with 25% probability. If *A*_*e*_ is not equal to *E*_*p*_, then, the agent has a worry, because both the neighbor’s influence and exits’ potential are against the current behavior. Since the major factor of panic does not exist, the agent would make a switch exit change with a 50% probability.

Before the behavior of the agent A in panic is described, it is, worthwhile, stating that A is already in a big worry due to panic. When A is in panic and *A*_*e*_ is equal to *N*_*e*_, it may exaggerate its worry due to neighbors also moving to the current exit, which seems counter-productive. Now, if *A*_*e*_ is equal to *E*_*p*_, this may give the agent a little relief and, hence, it would switch an exit with a 50% probability. When *E*_*p*_ is not in favor (means it’s not the same as *A*_*e*_), the agent would switch an exit with a 75% probability. If A is in panic and *A*_*e*_ equals *N*_*e*_, then, there is something to worry about. Now, if *A*_*e*_ is also the same as *E*_*p*_, then, the agent may decide to change the exit but with a little motivation. In this case, the agent would switch an exit with a 50% probability. Otherwise, the agent would be more motivated about changing the current exit with 75% probability. When A is in panic and *A*_*e*_ is not the same as *N*_*e*_, then, the agent has to worry a lot. If *A*_*e*_ is also not equal to *E*_*p*_, the agent would change the current exit with 75% probability, otherwise, it will definitely switch the exit.

 
_________________________________________________________________________________________________ 
  Algorithm 3: The Logic behind Strategy 4 (S4)                                ____ 
    Agent A is making a decision 
     Agent N is a random local agent 
     Agent M is agent with maximum mobility index (including both local and 
      distant agents) 
     Ae ← current exit of A 
     Ap ← current panic index of A 
     Ne ← current exit of N 
     Me ← current exit of M 
     if A is emotional then 
         if Ae! = Ne then 
       if Ap > 0 then 
   Ae = Ne [with probability 0.1] 
   end 
   end 
    end 
    if A is rational then 
         if Ap <= 0 then 
       if Ae == Ne then 
   Ae = Aalternate [with probability 0.1] 
   else 
   Ae = Aalternate [with probability 0.9] 
   end 
   else 
       Ae = Me 
   end 
    end    

#### Strategy 4 (S4)

Strategy S4 is the same as S2 with one exception that the alternate exit in S4 is the exit of one of the neighbouring agents, which has the highest Mobility Index (MI). The neighbouring agents include both local and distant neighbours. A long distant random link is established for each rational agent in this strategy. The logic of strategy S4 is given in Algorithm 3.

#### Differentiating the strategies in graphical form

**Figure 3:**
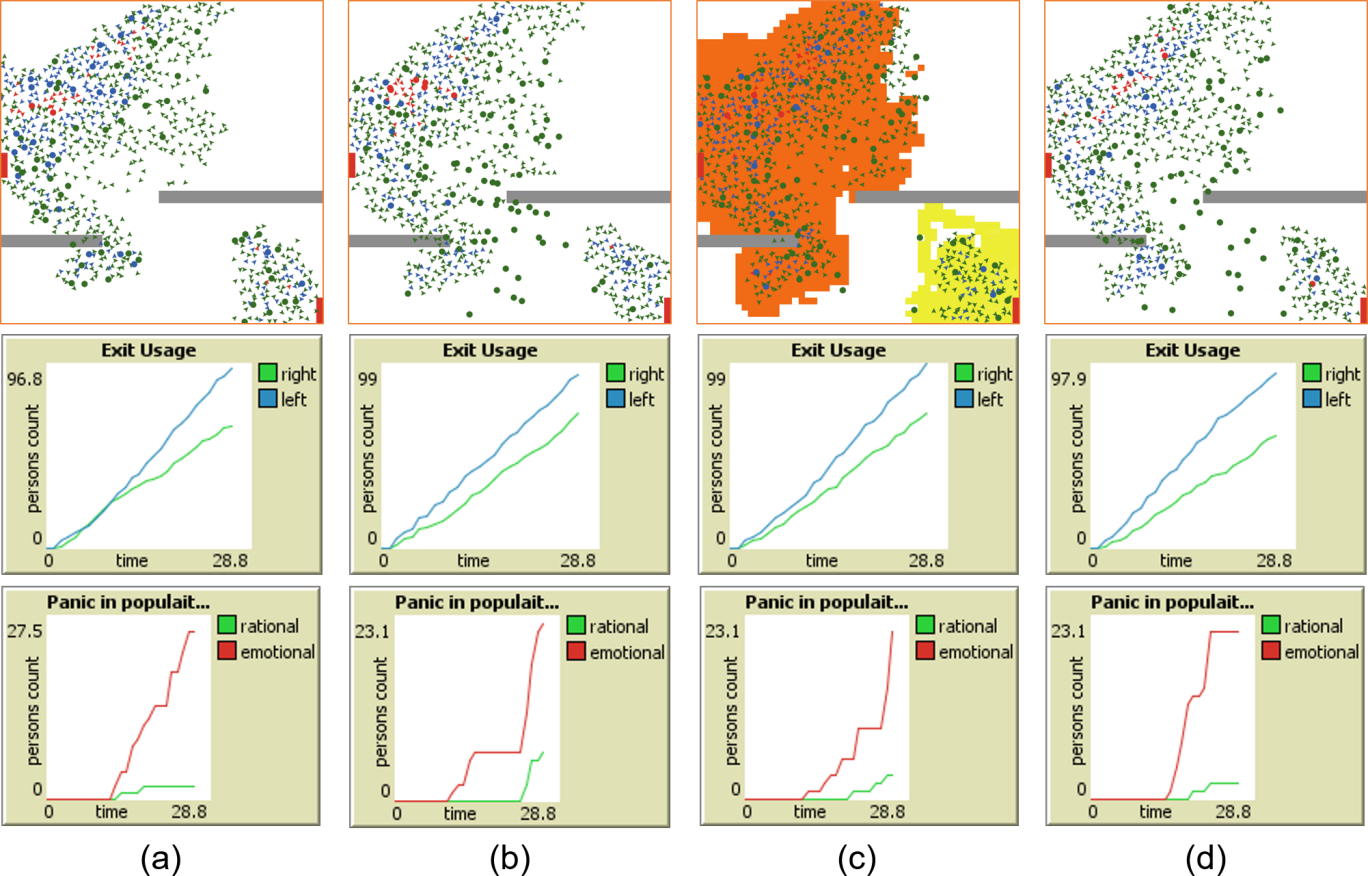
Screen-shots of the random simulation outcome at iteration 25, in case of (A) all agents only going to the nearest exit, (B) agents under the influence of locality driven rationality, (C) rational agents under the influence of social as well as potential field, and (D) rational agents under the influence of locality as well as distantly driven rationality. The accompanying graphs indicate the parameters for which the strategies are compared.

Figure 3 illustrates the differences between the four strategies along with the visualisation of the simulation space. It shows agents’ positions at iteration 25 for all the four situations. Rational agents are represented by circles while emotional by triangles. Different colors are used to represents various types of states, which are described later.

In Fig. 3A, all the agents are going only to the nearest exit, as directed by S1. Figure 3B shows the exit preferences of agents towards exits under the influence of locality driven rationality, as specified by S2. Exit preferences of rational agents under the influence of social as well as potential field in a rational way are provided in Fig. 3C, as proposed in S3. The emotional agents in this strategy follow the nearest exit. Figure 3D illustrates the exit distribution of rational agents under the influence of social rationality, as suggested by S4. In this case, a social circle include distant agents and emotional agents use the nearest exit.

The comparison of Fig. 3B and 3D and with Figs. 3A and 3C shows that quite a few rational agents moved from the nearest left exit to the right exit under the social influence. Detailed analysis of this will be presented in the following sections.

## ODD Protocol

Overview, Design concepts and Details (ODD) is a scientific protocol (Grimm, Polhill & Touza, 2017), used, for the documentation of ABM simulations. This section is dedicated to present, the work presented in this paper, in terms of ODD protocol.

### An overview

#### The purpose and patterns

The purpose behind the development of the proposed model is to evaluate the performance of an evacuating crowd from a hypothetical square-shaped space with two exits.

#### Entities, state variables and scales

Evacuees are the only agents. The state variables used by agents are: current exit, state, and MI. The current exit provides the exit an agent is currently heading to. The current cognitive state of an agent is represented by the “state” variable. Similarly, MI shows the current quality of mobility acquired by an agent. Patches use the state variables named: Structure_type, Doms, Hops, PVL, and PVR. The structure type shows the type of a patch being either normal (walkable), obstacle, or exit. The variable ‘Doms’ provides the directions of nearest paths for both left and right exits while ‘Hops’ maintains the distance (hop count) of nearest paths for both left and right exits. The PVL and PVR represent the potential field values for the left and right exits.

#### Process overview and scheduling

**Figure 4:**
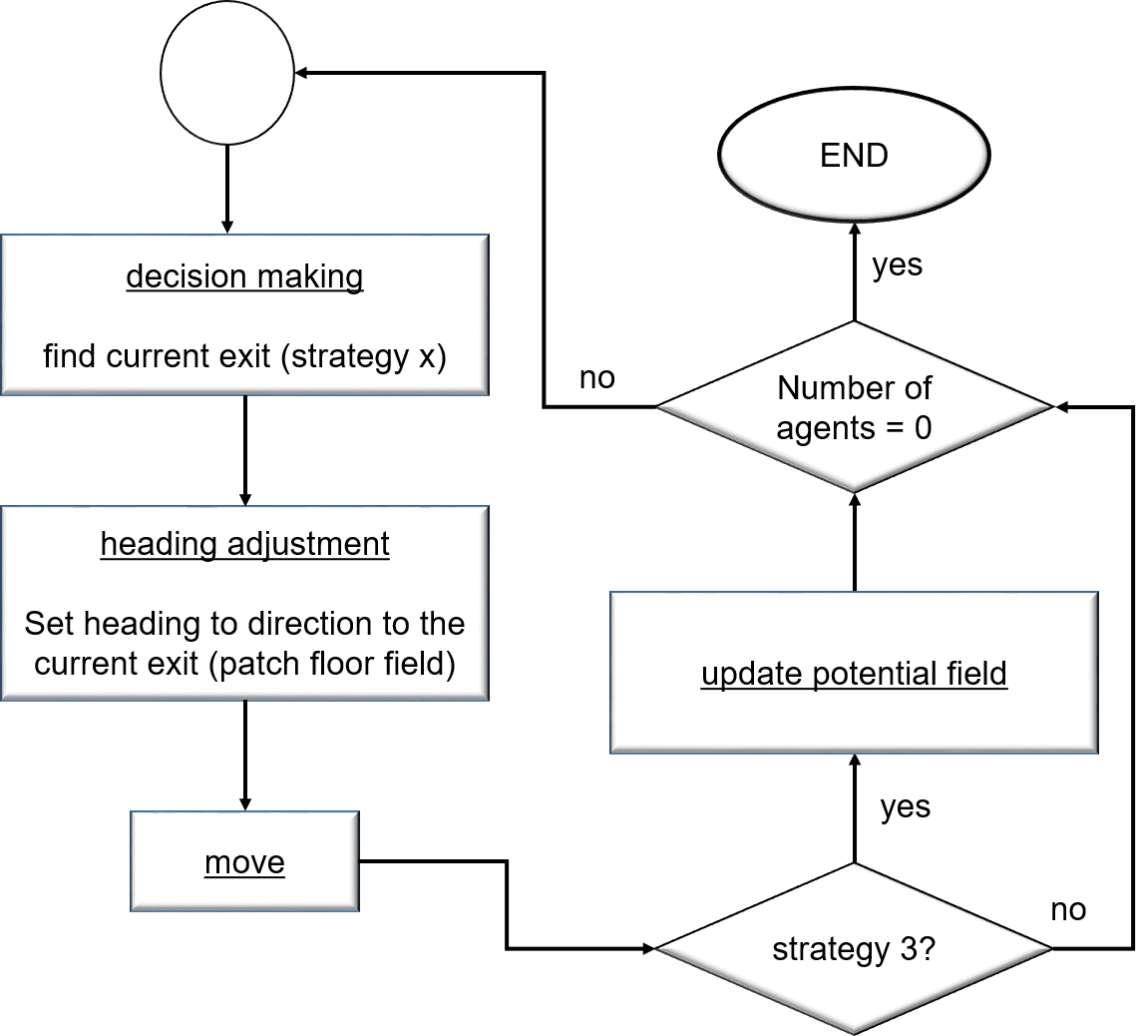
An abstract view of the simulation process and scheduling executed at each iteration by each agent.

Mobility and decision-making happens at each timestamp of the simulation, concurrently, for all agents. When an agent exits through one of the two exits, it is no more part of the simulation. The number of agents in the simulation is used for terminating the simulation. The procedure, therefore, counts the total number of agents in the simulated space at each timestamp before it starts the rest of the activities. The simulation is stopped when this agent count becomes zero. Otherwise, the **decision-making** process allows an agent to determine the current exit to follow by taking strategy **x** as an input. Strategy **x** refers to one of the four strategies, described earlier. Once an exit is selected, the agent uses the procedure **heading adjustment** to adjust its direction using the floor field information available at the cell level. The agent, then, moves, whose dynamics are explained shortly. If the simulation is run for strategy S3, the potential field information is also updated after the move. Figure 4 provides an abstract view of the flow between various activities of the simulation.

**Figure 5:**
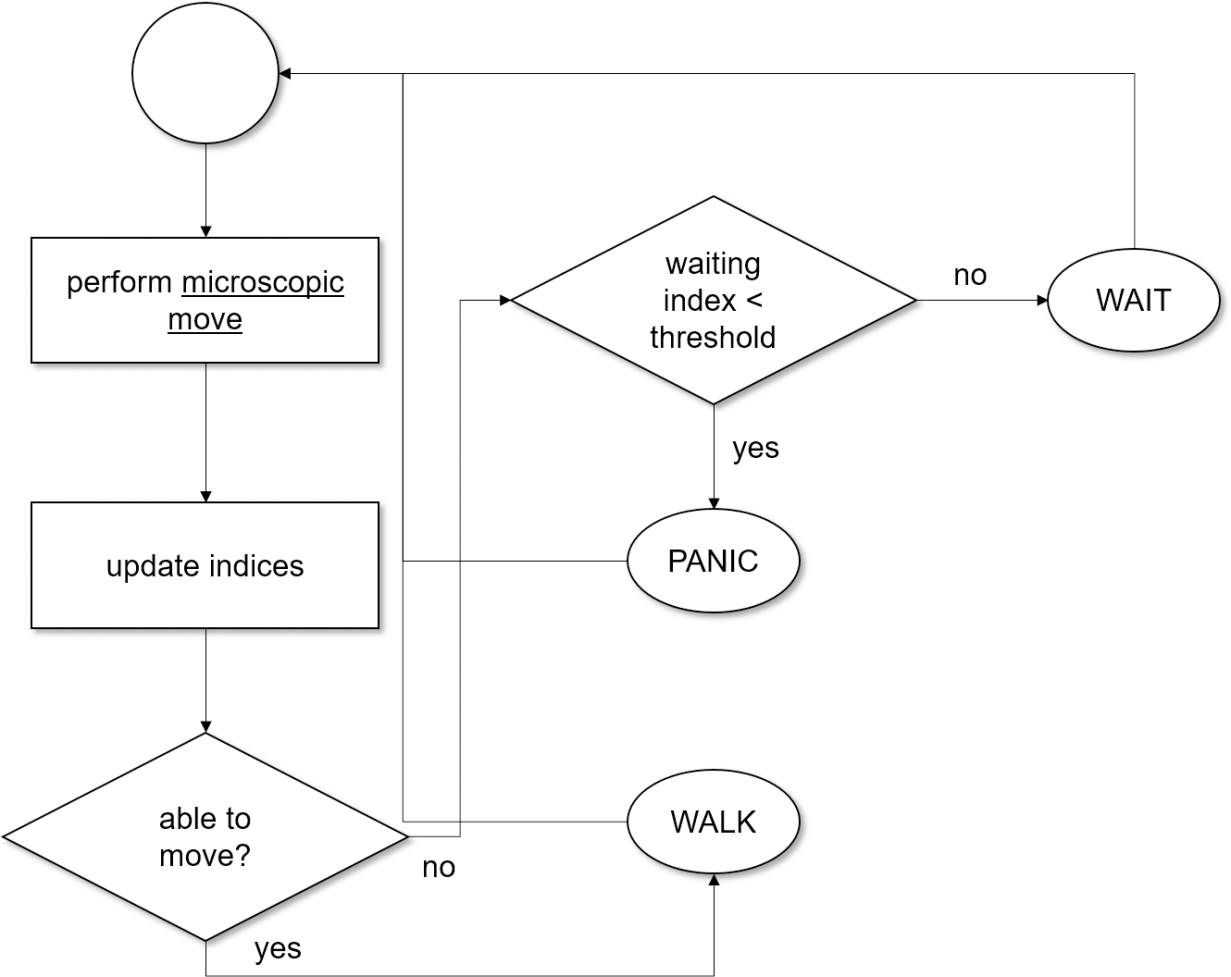
The detailed view of move process and state transition system executed at each iteration by each agent.

The initial state of agents is set to WALK. The agents perform microscopic move (described in ‘Space and time model’), concurrently, at each iteration. In response to a move, three indices (namely the moving, waiting and panic) get updated based on the current state of an agent. If the state of an agent is WALK, the moving index is incremented by 1 while the waiting and panic indices are reset to 0. If the state of an agent is WAIT, the waiting index is incremented by 1 while the rest of the two indices are reset to 0. Similarly, when the state of an agent is PANIC, the panic index is incremented by 1 while the other two indices are reset to 0. When the agent is able to move, it moves and sets its state to WALK for the next iteration. However, when it fails to move, it may enter either into WAIT or PANIC state depending on the waiting index. When the waiting index is less than a threshold, its state is set to WAIT, otherwise, it is set to PANIC. Figure 5 provides the detailed process of move operation and its related transitions. When an agent reaches an exit, it is deleted from the simulation. Global variables (which are detailed in ‘Initializations’ and used for reporting purposes) are updated at each iteration.

### Design concepts

#### Basic principles

The proposed model addresses the basic principle of using discrete time and space simulation for evacuation purposes. Different models of agents’ interaction are tested to perform a comparative analysis of various individual, informational and social behaviors in terms of evacuation efficiency. The basic objective behind the adaptive behavior of agents is to avoid panic and keep them moving.

#### Emergence

S1 is base strategy for evacuation, in which all the agents are moving towards the nearest exit. Strategies S2 to S4 use social and informational behavior for the emergence of a more distributed and less congested population of agents, with the help of actions of most of the rational agents, while evacuating a space with two exits. We believe that the situations/cases where the people are distributed as much uniform as possible among the two exits and when they are not panicking (actually a by-product of lesser congestion) will have fewer chances of a disaster. The purpose of the three strategies (S2 to S4) is to find conditions, which would let a favourable flow of evacuees to emerge.

#### Adaptation

The adaptation of behavior is a central part of the model. Rational agents adapt their behavior, in response to social pressure in the proximity and distant neighbors in S2 and S4. In S3, however, they adapt their behavior based on social proximic pressure as well as potential map diffused by exiting agents. Game-theoretic approach is adopted by all adaptation mechanisms, in which different situations are evaluated based on relative payoffs.

#### Sensing

Sensing plays a vital role in making exit decisions. To make rational decisions, agents need to sense the current state of the surrounding neighbors and their exit choices. In case of strategy S3, the agents require to get potential field information. Similarly, strategy S4, further, suggests sensing the MI and exit choice of a distant neighbor. In microscopic move, the agents sense their locality for possible collisions and avoid them by using the corresponding collision avoidance mechanism.

#### Interaction

Different types of interactions between the agents and patches such as agent to agent, agent to patch, patch to agent, and patch to patch enable various activities. They not only setup floor field for the nearest direction and distance to both exits but also help spreading and decaying informational content (the potential field) by agents and patches. They materialize social interaction among agents in the neighborhood and enable distant interaction among the agents. Furthermore, they support interactions between agents and environmental structure.

#### Observations

Simulation results are evaluated based on three quantitative measures described below:

1. Exit Time: collects the number of iterations (simulation ticks) needed by all agents to exit through the designated exit points.
2. Exit Usage: shows the number of agents, who left the system through two different exits.
3. Agents in Panic: collects the maximum number of agents in panic during the simulation.

### Details

#### Initialization

This work needs to set up patches, agents, and global reporting variables. Therefore, the initialisation is divided into three groups.

The default structural type of all the patches’ is normal (walkable). To setup exits, each exit patch is assigned a name: left and right in this work. To setup non-walkable obstacles, the environment type parameter obstacle is used. The last step is to create the floor field value of the patches.

Once the patches are initialized, we initialize agents as follows. All agents are randomly assigned to unique walkable patches without overlapping. The moving, waiting and panic indices are, then, all set to 0. The current exit for all agents is set to the nearest exit, which are collected from the ‘Hops’ variable of their underlying patches. The state of all agents is set to WALK and their type is set to emotional while the type of required number of agents is set to rational.

This work uses a number of global reporting variables and they are all initialized to 0. They are the tick counter indicating the total simulation time, the number of agents who exit from left and right exits, and the number of rational and emotional agents who enter into the PANIC state at some time during the simulation.

#### Sub models

Three types of sub-models are used: update Update Mobility Index,, Spread and Decay, in this work.

**update MI**: Mobility Index (MI) represents the quality of mobility, obtained by an agent, with the passage of time. Initialized with a value of 0, MI is updated at each iteration based on the current state of the agent, as follows. It is incremented with a 1 when the current state is MOVE and decremented with a 1 when the state is WAIT. MI = MI − (MI × sensitivity) when the current state of the agent is PANIC. Sensitivity has a static 0.5 value. MI value is used in S4, in which, it is required to have an agent M with the highest MI value in the neighborhood of a rational agent.

**spread**: This sub-model is responsible for spreading the potential field of an exit (*PV*_*X*_). When an agent reaches an exit, the patch variable *PV*_*X*_ of all neighboring patches is set to 1, where X represents both left and right exits. For other agents, if a patch (say P) on which an agent is residing, has *PV*_*X*_ greater than 0, the list of all neighboring patches would be potentially updated. For every neighboring patch N, if its *PV*_*X*_ is less than *PV*_*X*_ of P, *PV*_*X*_ of N will be decremented by a fraction (one-tenth of *PV*_*X*_ of P). Hence, starting from the potential field value of 1, the exit announcements would be spread out with lesser and lesser intensity, provided that there is an agent encountered.

**decay**: This sub-model just lets the potential value of patches decay with the passage of time.

## Simulation and Results

### Simulation setup

This work is simulated with a 2D bounded grid space that comprises 51 × 51 cells. The simulation starts with a number of agents in the space and stops when the space becomes empty. However, the density, in this work, is kept static to a value of 1,000. The simulated space has two exits named left and right. This study investigates different scenarios based on three parameters: Environment Type, Strategy, and % of rational population. Thus, each case is made up of the tuple [Environment type, strategy, and % of rational agent in the population]. It uses four different types of environments namely E1, E2, E3, and E4, which correspond to Figs. 2A to 2D, in the given order. Four different evacuation strategies S1, S2, S3 and S4 are used, which are explained in detail in ‘Models’. This work uses four different percentages (5%, 10%, 15%, and 20%) of rational agents among the total population. Every simulation case is repeated 50 times and their average values are then used for comparison purposes.

### Simulation results

#### Exit time

**Figure 6:**
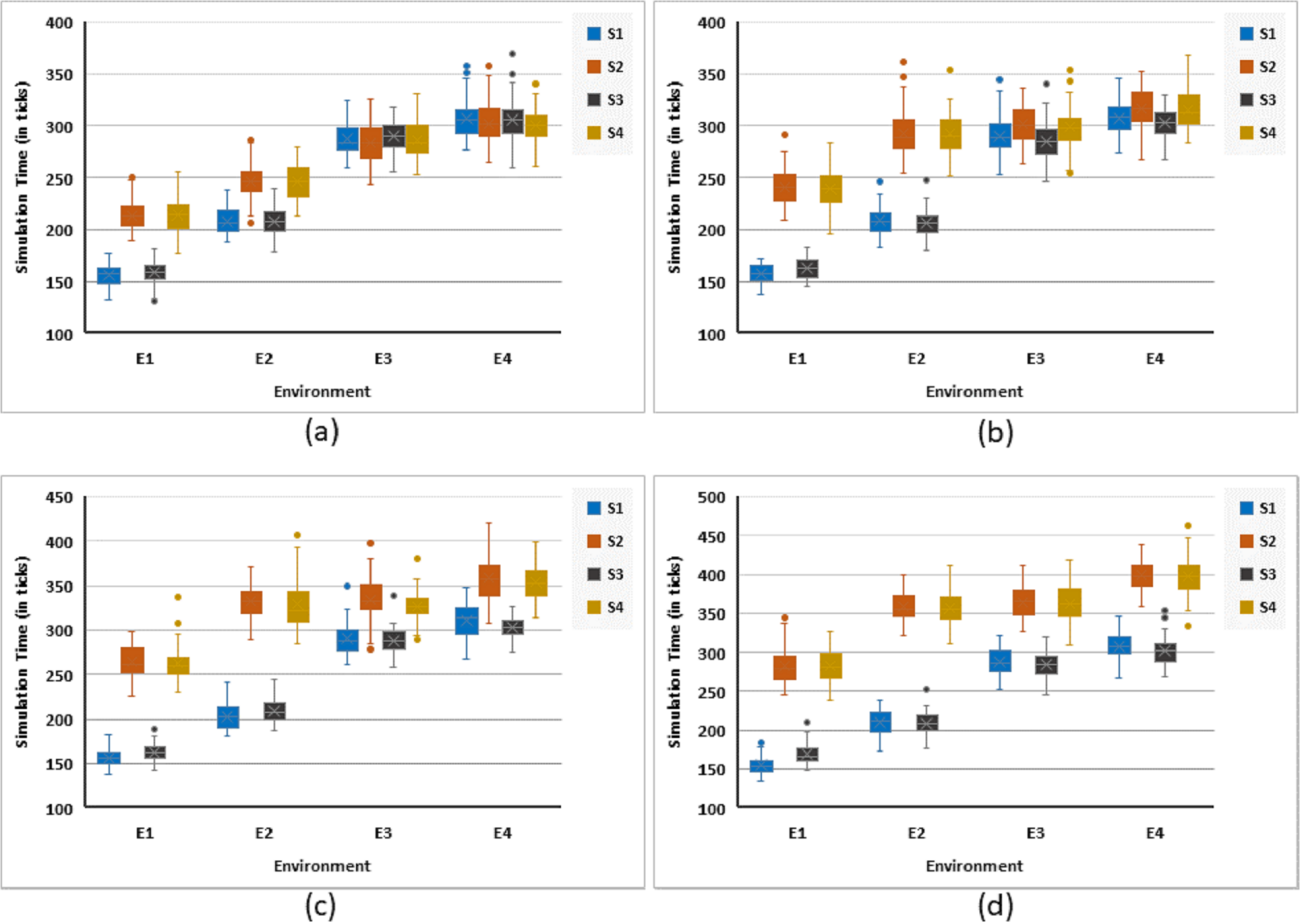
Simulation time (in terms of ticks) for all strategies against four different environments. Illustrating simulation responses for: (A) 5%, (B) 10%, (C) 15%, and (D) 20% rational agents.

Figure 6 presents simulation results for different scenarios. It shows that the exit time, generally, increases with an increase in the complexity of the environment. However, the comparative analysis of strategies suggests grouping S1 and S3 as one while S2 and S4 as the second group. The former group is much more efficient (in terms of exit time of the population) than the latter. However, this difference vanishes in more challenging environments, where the number of rational agents is only 5% of the total population. As the percentage of rational agents increases, the former group starts to become more and more efficient.

In spite of the fact that S1 is the best (in terms of exit time) as all the agents are directed towards the nearest exit, irrespective of their panic and emotional state, S3 is comparable to it. In fact, it is slightly better in complex environments. Redirection of agents to alternate exit, in the response to social influence, definitely increases the exit time of the population. However, it must be emphasized that in many situations, for a herding crowd, the factor of panic in people can be a more important factor than exit time. When S2 is compared with S4, minor improvements are observed in some settings, however, they can be ignored. Though the effect of population density on exit time is not relevant here, however, it was observed in our previous work (Zia & Ferscha, 2020) that exit time increases with population density.

#### Exit usage

**Figure 7:**
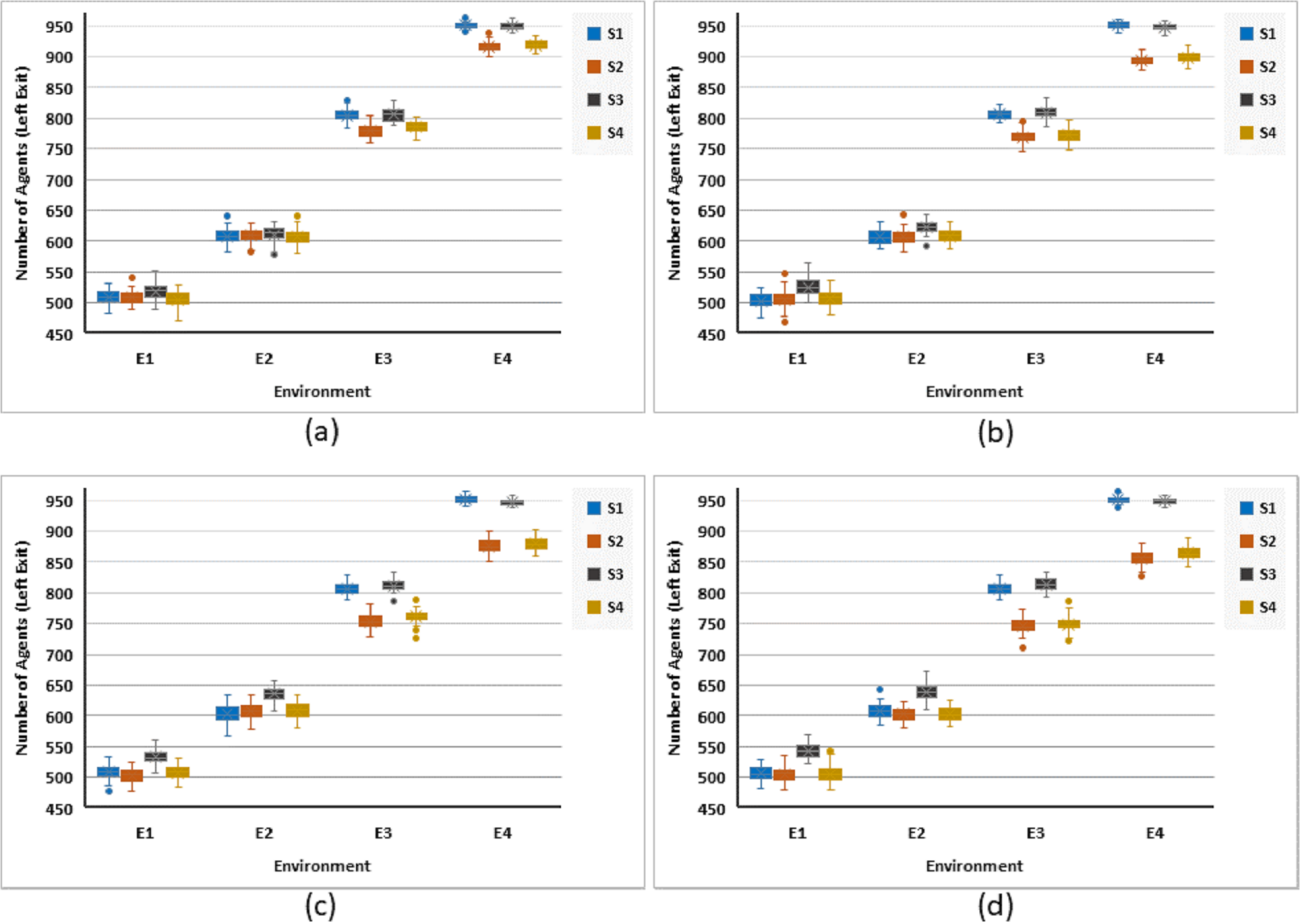
Number of agents, who exit the space through the left exit for all strategies against four different environments. Illustrating simulation responses for (A) 5%, (B) 10%, (C) 15%, and (D) 20% rational agents.

Quantitatively, the reason of the exit time, being good or bad, is due to the change of agents’ preferences regarding the exit. Based on the geometric consideration of simulation space, the exit time is good, when many agents change their exit from left to right. The columns in Fig. 7 show the number of agents actually ended up at the left exit for various scenarios, at the end of the simulation. We seek to have a lesser and lesser number here, which is equivalent to equal distribution of agents across both exits. However, the distribution of exit usage among left and right exits, generally deteriorates, with an increase in the complexity of the environment. The comparative analysis of strategies suggests grouping S1 and S3 into one while S2 and S4 into the second group. The former group is less efficient (in terms of agents’ distribution across the exits) than the latter. The difference between the two groups is more significant in more challenging environments. It can also be seen that S2 performs marginally better than S4.

More equal distribution of agents across both exits is expected to decrease the panic in the population, which is analyzed next.

#### Agents in panic

**Figure 8:**
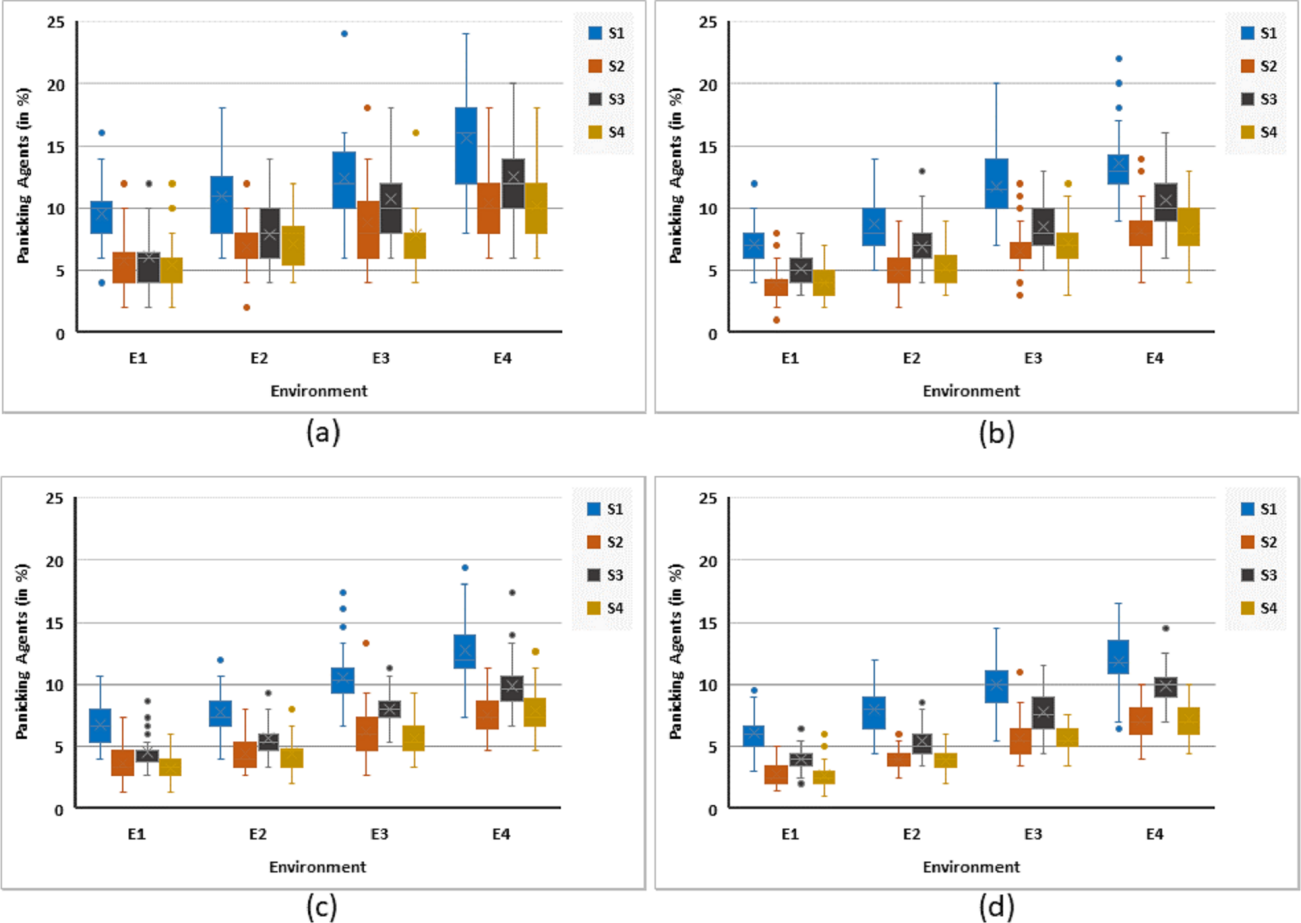
Maximum %age of rational agents in panic for all strategies against four different environments. Illustrating simulation responses for (A) 5%, (B) 10%, (C) 15%, and (D) 20% rational agents.

Reduction of panic among the crowd was one of the main purposes of this work. Figure 8 illustrates simulation trends for various cases based on different strategies, environments, and rational agents. It is clear that S3 has always less number of panicking agents as compared to S1. However, the number of panicking agents increased with an increase in the complexity of an environment. It can be observed that S1 performs the worst followed by S3 in all cases irrespective of the percentage of rational agents among the population. Strategies S2 and S4 have a similar and improved performance (against both S1 and S3) as they have a reduced number of agents in panic during all the cases. The comparative analysis of these strategies suggest placing S1 and S3 in one group while S2 and S4 in the second group. The former group is less efficient, in terms of panicking agents, than the latter one. Though, the difference between the two groups is consistent in all cases, however, it is more obvious in complex challenging environments. In general, S2 performs very similar to S4 in all cases.

## Discussion

The main objective of the development of different strategies was seeking a possible emergence of favorable situation for an evacuating crowd at a global level, where people are either emotional or rational, and the environment has different levels of complexity. The results are evaluated based on three measures: (i) time to exit, (ii) distribution of exits’ usage, and (iii) population in panic.

S1 is the base case but it was supposed to perform the best in terms of exit time. Ignoring the case, where the %age of rational agents is really low (5%), S1 was always better than other strategies but specifically in the case of simple environments. S3 was also not that far from S1 in all situations. Moreover, S1 was supposed to perform the worst in terms of exits’ usage and panic. It turned true, especially, for the complex environments. S3 was again very similar to S1 in all these cases. However, S3 performed, consistently, much better than S1 in terms of panic in all cases. Hence, it can be inferred that strategy S3, which relies on rich informational content to be transmitted through potentially peered ICT devices, are not of great use, in general. However, it could be beneficial in those evacuation scenarios, in which the situation is not that dangerous and it is performed in a structured way, where the people know each other. This typically applies to residential buildings, factories, and offices with complex geometries and where there is no disastrous situation such as some chemical leakage, which is not that harmful. In fact, the success of S3 depends on this as people without an apparent herding situation would most probably be able to pay attention to the information available to them. In other words, strategy S3 can be applied in those situations, where we are confirmed that people would behave rationally and would not lose their temper (as a whole). It can be facilitated by providing information such as ’exit to take’, in form of announcements and exit signs, to the evacuees with the help of some technology-based on exit dynamics and knowledge about their impact.

Panic is related to the complexity of the environment. When the geometry of the environment is more complex, there would be more panic. It is reiterated here that a person gets panic when he/she is unable to proceed towards one of the exits for a longer period of time. The complexity of the environment, therefore, makes the panic issue severe. The people in the strategies S2 and S4, which are social in nature, get less panicked. S2 is just based on human behavior, whereas, S4 also considers distant connectivity.

It has been observed that people do ask for help from their distant friends when they get stuck in an emergency situation. Results have suggested that the response of a simple mechanism of doing against what others are doing, in a panic situation, emerges in a much better shape. However, this is against the psyche of a herd. Still, people may be trained not to be in a panic and act rationally to give them more chances of survival. It was learnt that S4 has no substantial advantage over S2. This might be due to the nature of environmental structures used in this work. It, however, could turn out to be advantageous over S2, when tested against more complex and dynamically changing environments. But, this has yet to be seen and left as a future work.

## Conclusion

This paper proposed an agent-based model, providing a framework, to explore individual, social and technological aspects of crowd evacuation. It combined many features, which are related to an evacuating crowd and they are categorized as individual, social and technological features. Models of these (called strategies) were pivoted onto a common agent-based modeling framework and a grid-based hypothetical environment. By simulating these models, an insight into the effectiveness of several interesting evacuation scenarios is provided.

Simulation results revealed that there is no substantial advantage of informational overload on a crowd in an evacuation. This may only marginally work in favor of people, and probably only in those situations, in which there are fewer chances of herding. In more serious situations, people should be left alone to decide. But, they can be taught not to panic during the drills so that they are better able to make their decisions, solely based on the dynamics of their surroundings. Also, distant connectivity has no apparent advantage as well. But, this can well be due to the nature of the environmental structures that were used. With more complex and dynamically changing environments, information coming from distant ties may turn out to be vital.

Limitations: Although the behavioral assumptions about people in panic during a herding situation have an underpinning on behavioral theories, empirical evidences and models progression but building blocks of human behavior and environmental dynamics used to develop the model, in this paper, are not explicitly verified.

##  Supplemental Information

10.7717/peerj-cs.531/supp-1 Supplemental Information 1 The source code of evacuation model in NetLogo The NetLogo model developed to evaluate the work presented in this paper.

## Additional Information and Declarations

### Data Availability

The following information was supplied regarding data availability: The code of an agent-based model developed using NetLogo is available in the Supplementary File.
